# Toxin ζ Triggers a Survival Response to Cope with Stress and Persistence

**DOI:** 10.3389/fmicb.2017.01130

**Published:** 2017-06-23

**Authors:** María Moreno-del Álamo, Mariangela Tabone, Virginia S. Lioy, Juan C. Alonso

**Affiliations:** Department of Microbial Biotechnology, Centro Nacional de Biotecnología (CSIC)Madrid, Spain

**Keywords:** toxin-antitoxin system, cell wall inhibition, c-di-AMP, CodY, (p)ppGpp, DisA

## Abstract

Bacteria have evolved complex regulatory controls in response to various environmental stresses. Protein toxins of the ζ superfamily, found in prominent human pathogens, are broadly distributed in nature. We show that ζ is a uridine diphosphate-N-acetylglucosamine (UNAG)-dependent ATPase whose activity is inhibited *in vitro* by stoichiometric concentrations of ε_2_ antitoxin. *In vivo*, transient ζ expression promotes a reversible multi-level response by altering the pool of signaling purine nucleotides, which leads to growth arrest (dormancy), although a small cell subpopulation persists rather than tolerating toxin action. High c-di-AMP levels (absence of phosphodiesterase GdpP) decrease, and low c-di-AMP levels (absence of diadenylate cyclase DisA) increase the rate of ζ persistence. The absence of CodY, a transition regulator from exponential to stationary phase, sensitizes cells to toxin action, and suppresses persisters formed in the Δ*disA* context. These changes, which do not affect the levels of stochastic ampicillin (Amp) persistence, sensitize cells to toxin and Amp action. Our findings provide an explanation for the connection between ζ-mediated growth arrest (with alterations in the GTP and c-di-AMP pools) and persistence formation.

## Introduction

The toxin-antitoxin (TA) systems are widely distributed in free-living bacteria, in their extrachromosomal elements, and in archaea (Gerdes, 2013; Unterholzner et al., 2013). The toxins of all known TA systems are proteins while the antitoxins are either proteins or non-coding RNAs. The TA systems are classified into five different TA types (Yamaguchi et al., 2011), being the most broadly distributed the type II TA system, where both the toxin and the antitoxin are proteins (Leplae et al., 2011; Gerdes, 2013). The type II toxins use different strategies to regulate growth control and cellular processes related to the general stress response. Toxins of the ζ/PezT superfamily, which are among the most broadly distributed in nature, are found in major human pathogens and in environmentally important bacteria of the phylum Firmicutes (Mutschler and Meinhart, 2013). The plasmid-borne ζ gene product from *Streptococcus pyogenes, Streptococcus agalactiae* or *Enterococcus faecalis* and the chromosome-encoded ζ toxin from *Clostridium perfringens* or *Staphylococcus aureus* (~285 amino acids) share ~43% sequence identity with chromosome-encoded *Streptococcus pneumoniae* or *Streptococcus suis* PezT toxin (~255 amino acids) (reviewed in Mutschler and Meinhart, 2013). When free in solution, these toxins interact with uridine diphosphate-N-acetylglucosamine (UNAG), ATP-Mg^2+^ or GTP-Mg^2+^ (denoted ATP and GTP), and with their cognate dimeric ε/PezA antitoxin (ε/PezA_2_) (Meinhart et al., 2001, 2003; Khoo et al., 2007; Mutschler et al., 2011). A non-toxic heterotetrameric complex (ζε_2_ζ/PezT-PezA_2_-PezT) interacts with UNAG, but not with ATP/GTP (Meinhart et al., 2001, 2003; Khoo et al., 2007; Mutschler et al., 2011).

Enzymes of the ζ/PezT toxin superfamily have a common fold core with phosphotransferases (Meinhart et al., 2001, 2003; Khoo et al., 2007; Mutschler et al., 2011). Toxin ζ/PezT transfers the ATP/GTP γ-phosphate to the 3′-hydroxyl group of the UNAG amino sugar, rendering UNAG-3P unreactive and thus reducing cell wall biosynthesis (Mutschler et al., 2011). Although, a quantitative analysis of this reaction showed that in the presence of limiting UNAG and ATP, toxin ζ mainly hydrolyzed ATP and only traces of the γ-phosphate are transferred to UNAG (Tabone et al., 2014a).

The fine mechanisms of bacterial responses to toxin action are not generally conserved among different bacterial phyla (Gerdes, 2013). The evolutionary distance between *Escherichia coli* and *Bacillus subtilis*, which is larger than the time divergence between yeasts and humans, reflects the notable differences made by the purine nucleotides in the stringent response (Potrykus and Cashel, 2008; Liu et al., 2015). In *E. coli* (a representative of the γ-proteobacteria class), toxin-mediated persister formation is linked to high levels of guanosine (penta)tetraphosphate ([p]ppGpp), which inhibits the PPX phosphatase; dropping of PPX increases polyphosphate levels that activate Lon protease degradation of the antitoxins, with subsequent release of active toxins (Maisonneuve et al., 2013). These free mRNase toxins contribute to persistence to some, but not all antibiotics (Harms et al., 2016). The role of toxin action in bacteria of the phylum Firmicutes, and whether these toxins induce persistence or tolerance, is poorly understood. We therefore examined the role of *S. pyogenes* pSM19035-encoded ζ toxin in growth arrest (dormancy), alone or with antibiotic in *B. subtilis* cells (representative of the Firmicutes), by controlling expression of the toxin at or near physiological concentrations. In our analysis, we did not study the role of (p)ppGpp in antitoxin degradation and free toxin release. We found that transient expression of a short-lived toxin ζ variant (ζY83C) induced different temporal sets of cell responses and growth arrest, but a small cell subpopulation (5 × 10^−5^ to 1 × 10^−4^) exits the dormant state, leading to persistent or tolerant *B. subtilis* cells (Lioy et al., 2012).

Analysis of the metabolic changes induced by the free toxin showed that within the first 5 min, ζY83C expression decreased the intracellular GTP pool and dysregulated transcription of 78 genes, of which 28 with reduced expression are essential for cell proliferation (Lioy et al., 2012). Induction of genes involved in the SOS response was not observed, but the expression was documented of genes that could modulate toxin action, such as increased *comGA* and *relA* expression or decreased *glmS* gene expression (Lioy et al., 2012). It is likely that by altering ATP:GTP ratios, toxin ζY83C modifies availability of the initiating nucleotides; this in turn changes promoter preferences by RNA polymerase, and the intracellular signaling (Krasny and Gourse, 2004; Pedley and Benkovic, 2017).

Within the first 15 min of ζY83C expression, the intracellular ATP concentration decreases and that of (p)ppGpp increases (Lioy et al., 2012). The contribution of increased *comGA* and *relA* expression lead to higher (p)ppGpp levels (Potrykus and Cashel, 2008; Hahn et al., 2015; Liu et al., 2015), which directly inhibit both salvage and *de novo* GTP synthesis (Lopez et al., 1981; Kriel et al., 2012; Pedley and Benkovic, 2017). In *B. subtilis*, low GTP levels lead to derepression of CodY, a global transcriptional regulator from exponential to stationary phase (Handke et al., 2008; Kriel et al., 2012; Bittner et al., 2014; Brinsmade et al., 2014).

Downregulation of GlmS contributes indirectly to reducing the pool of UNAG synthesis, and a small UNAG pool increases levels of the essential cyclic 3,5-diadenosine monophosphate (c-di-AMP) second messenger (Witte et al., 2008; Zhu et al., 2016). Changes in the intracellular level of c-di-AMP, which play an essential role in K^+^ transport and cell wall homeostasis (Gundlach et al., 2017), indirectly increase the intracellular (p)ppGpp pool (Rao et al., 2010; Corrigan et al., 2015). The relationship between the effective levels of c-di-AMP and bacterial persisters is nonetheless poorly characterized.

At later stages of toxin ζY83C expression, synthesis of macromolecules (DNA, RNA, proteins) is inhibited and membrane potential is impaired (30–90 min; Lioy et al., 2012). Direct interaction of (p)ppGpp with DNA primase inhibits DNA replication (Wang et al., 2007; Srivatsan and Wang, 2008), (p)ppGpp-mediated low levels of GTP decrease mRNA transcription (Krasny and Gourse, 2004), and the essential GTPases decrease the amount of mature 70S ribosomes and reduce translation (Corrigan et al., 2016). Within 60–120 min, cell wall biosynthesis is reduced by ζ-mediated phosphorylation of a UNAG fraction, leading to accumulation of unreactive UNAG-3P (Mutschler et al., 2011; Lioy et al., 2012), and by (p)ppGpp inhibition of peptidoglycan metabolism (Eymann et al., 2002). All these metabolic changes are reversible, however, because when the stress condition is relieved (or after artificial induction of antitoxin expression), the antitoxin ε_2_ reverses the ζ-induced dormant state and the cell population “awakens” (Tabone et al., 2014a,b).

**Figure 1:**
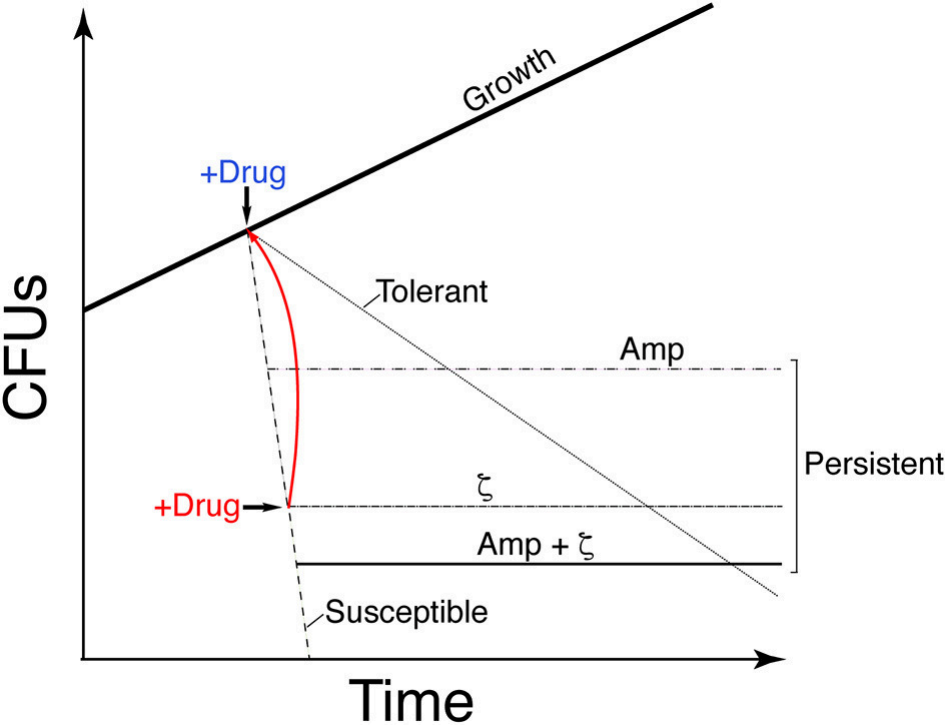
Graphic illustration showing the difference in growth of the different stress survival strategies. Proliferation of susceptible clonal cells is halted by transient toxin ζ expression (IPTG addition) or Amp addition (2x MIC) (+Drug, blue). A large fraction of cells is susceptible to the drug (dashed line); a subpopulation persists and forms colonies, leading to a biphasic time-inactivation curve (ζ [dotdashed] or Amp [twodotted dashed] persisters) rather than a linear time-inactivation curve (tolerants; dotted line). Transient expression of antitoxin ε_2_ (+Drug, red) awakens the susceptible cells to toxin ζ action (solid red line). Transient toxin ζ expression and Amp addition yield distinct persister subpopulations.

When bacterial growth is challenged by addition of antibiotic, susceptible cells stop growing, but a small subpopulation shows persistence (a biphasic time-inactivation curve) or tolerance to the drug (a linear time-inactivation curve; see Figure 1; Lewis, 2010; Amato et al., 2013; Brauner et al., 2016). These complex phenotypes have been attributed to diverse stochastically induced stresses, with the toxin reducing the activity of the antibiotic or enhancing efflux activities to form persisters or tolerant cells (Lewis, 2010; Balaban et al., 2013; Brauner et al., 2016; Harms et al., 2016) or to produce cells susceptible to antibiotic action, as in *B. subtilis* (Wu et al., 2011; Tabone et al., 2014b). Toxin ζ increases (p)ppGpp and decreases GTP pools, thus decreasing antibiotic persistence/tolerance formation; in contrast, low, dysregulated (p)ppGpp levels (in the Δ*relA* context) increase toxin and antibiotic persistence/tolerance (Tabone et al., 2014b).

To analyze how toxin ζ helps to induce a growth arrest state (dormancy), how antitoxin ε_2_ promotes exit from this state, and to learn about the interconnection between toxin action and the persister/tolerant state, we have studied the metabolic activities of purine nucleotides on persister/tolerant bacterial. Transient controlled expression experiments with toxin and antitoxin showed that toxin ζ induced a reversible growth-arrested state in a large fraction of proliferating, susceptible *B. subtilis* cells, but that a small subpopulation persists rather than tolerating toxin action (see Figure 1). Controlled upregulation of antitoxin ε_2_ reversed growth arrest *in vivo* and inhibited the UNAG-dependent ATPase activity of toxin ζ *in vitro*. GdpP- or DisA-dependent alteration of the c-di-AMP pool and CodY-dependent responses revealed that ampicillin (Amp) persisters and ζ-mediated persisters are distinct subpopulations, perhaps with different exit control, and that Amp enhanced killing of ζ-mediated persisters.

## Materials and methods

### Bacterial strains and plasmids

The bacterial strains and plasmids used in this study are listed in Table 1. All *B. subtilis* strains are isogenic with BG214. *Escherichia coli* BL21(DE3) cells harboring pBT290-borne ε gene under the transcriptional control of the T7 RNA polymerase-dependent promoter (*P*_T7_), or pCB920-borne wild type (*wt*) ζ gene under the control of *P*_T7_ and ε gene under its native RNA polymerase σ^A^-dependent promoter (*P*_ω_) were used for protein purification as described (Camacho et al., 2002; Tabone et al., 2014b).

**Table 1:** Bacterial strains.

**Strains**	**Relevant genotype**	**References**
BG1125^a^^,^^b^	+ *lacI, P*_hsp_ ζ, *aadA* [pCB799-*xylR, P*_xylA_ ε, *cat*]	Lioy et al., 2006
BG689^a^	+ *xylR, P*_xylA_ ζY83C, *cat*	Lioy et al., 2006
BG1145^a^	+ Δ*relA, xylR, P*_xylA_ ζY83C, *cat*	Lioy et al., 2012
BG1325^a^	+ Δ*gdpP, xylR, P*_xylA_ ζY83C, *cat*	This study
BG1323^a^	+ Δ*disA, xylR, P*_xylA_ ζY83C, *cat*	This study
BG1525^a^	+ *codY*::(*erm*::*spc*), *xylR, P_xylA_*ζY83C, *cat*	This study
BG1527^a^	+ *codY*::(*erm*::*spc*), Δ*disA, xylR, P*_xylA_ ζY83C, *cat*	This study
BL21(DE3)^c^	+ [pCB920, *P*_T7_ ζ gene, *P*_ω_ ω and ε genes, *bla*]	Tabone et al., 2014a
BL21(DE3)^c^	+ [pBT290, *P*_T7_ ε gene, *bla*]	Camacho et al., 2002

a *All Bacillus subtilis strains are isogenic with BG214 (trpCE metA5 amyE1 ytsJ1 rsbV37 xre1 xkdA1 att^SP^ att^ICEBs1^)*.

b *BG1125 cells bearing pCB799-borne ε gene were grown in MMS7 medium containing 0.05% xylose to titrate basal expression of the wt ζ toxin*.

c *Escherichia coli BL21(DE3) genotype (ompT gal [λ DE3, int::lacI::Plac_UV5_::T7 gene 1] fhuA2 [dcm] ΔhsdS)*.

### Growth conditions

The BG214 derivatives were grown to mid-exponential phase (~5 × 10^7^ cells ml^−1^) at 37°C in minimal medium S7 (MMS7) supplemented with the necessary amino acid (Lioy et al., 2006). Except for Δ*relA*, strains were grown in MMS7 with methionine and tryptophan at 50 μg ml^−1^ each (Lioy et al., 2006). The Δ*relA* strain shows an “auxotrophy phenotype” for valine, leucine, isoleucine and threonine, and was also supplemented with these amino acids (25 μg ml^−1^ each) (Roche, Germany; Lioy et al., 2006).

BG1125 bearing *lacI*-*P*_hsp_ *wt* ζ and pCB799-borne *xylR*-*P*_xylA_ *wt* ε (Table 1), in which ζ gene expression (transcribed by *P*_hsp_) is regulated by IPTG (Calbiochem, Spain) addition and the ε gene (transcribed by *P*_xylA_) is regulated by xylose (Xyl, Sigma, USA) addition (Lioy et al., 2012), was grown in MMS7 supplemented with Xyl (0.05%). In the absence of IPTG [Sigma, USA] there are ~40 ζ toxin monomers/colony-forming units (CFU), which lead to genetic rearrangement. To titrate basal ζ toxin levels, traces of Xyl (0.05%) were added to allow synthesis of low but marked ε_2_ antitoxin levels by the pCB799-borne ε gene. After IPTG addition, toxin ζ concentration increased in a very short time (10 min) up to ~1,500 ζ monomers/CFU, and its steady-state level remained for at least 240 min; these toxin levels are considered the “physiological concentration” (Lioy et al., 2012). At indicated times, 0.5% Xyl was added to induce antitoxin ε_2_ expression, and the culture was incubated 15 min before being plated without inductor or with 0.5% Xyl (Lioy et al., 2012).

In BG689 or BG1145 bearing the *xylR*-*P*_xylA_ ζY83C cassette (Table 1), expression of the toxin ζY83C variant was induced by addition of 0.5% Xyl. BG689 or BG1145 cells were grown in MMS7 to ~5 × 10^7^ cells ml^−1^ at 37°C. Xylose addition increased ζY83C levels to a plateau within the first 10 min, and the steady-state level of the toxin remained for at least 240 min (Tabone et al., 2014b).

Where indicated, toxin and/or antitoxin expression was induced by adding IPTG and/or Xyl. Before plating, cells were centrifuged and resuspended in fresh LB medium to remove the inductor or the antibiotic, and dilutions were plated on LB agar plates containing glucose (which switches off *xylR*-*P*_xylA_ cassette expression) or Xyl to express the ε_2_ antitoxin. The survival rate was derived from the number of CFU in a given condition relative to CFU of the non-induced/non-antibiotic-treated control. Except Δ*relA*, cells grew in MMS7 with a doubling time of 50–60 min. The doubling time of Δ*relA* cells increased 1.4-fold compared to the BG689 strain. Normal-sized and small colonies were observed in the Δ*relA* and Δ*disA codY* contexts. All plates were incubated for 20 h at 37°C.

The minimum inhibitory concentration (MIC) of Amp [Sigma, USA] was estimated by exposing 1–3 × 10^6^ cells ml^−1^ (16 h, 37°C) in MMS7 with shaking (240 rpm). The Amp concentration used (3 μg ml^−1^) was twice the MIC (2x MIC). In the absence of inducer, the presence of the ζY83C (BG689 strain) or the ζ gene (BG1125 bearing pCB799) does not affect the MIC (Tabone et al., 2014b).

### Protein purification and biochemical assays

The *S. pyogenes* pSM19035-encoded ζ gene was overexpressed in *E. coli* BL21(DE3) cells from a rifampicin-resistant T7 RNAP-dependent promoter as reported (Tabone et al., 2014a). In short, IPTG was added to induce the expression of T7 RNAP that transcribed *wt* ζ toxin, and 30 min later rifampicin (Fluka, USA), was added to selectively block the expression of the ω and ε genes. After 120 min of incubation and full decay of the ε_2_ antitoxin, the cells were harvested. The over-expressed long-living ζ toxin was purified in two steps as described (Tabone et al., 2014a). The fractions containing the ζ protein were dialyzed against buffer A (50 mM Tris-HCl pH 7.5, 80 mM NaCl) containing 50% glycerol and stored at −20°C. The ε gene was overexpressed in *E. coli* BL21(DE3) cells harboring pBT290 under the control of rifampicin-resistant *P*_T7_ (Ceglowski et al., 1993), and antitoxin ε_2_ was overexpressed, and purified as described (Camacho et al., 2002). The purified protein was stored in buffer A containing 50% glycerol at −20°C (Camacho et al., 2002).

The ATPase, dATPase or GTPase activities of ζ toxin were measured using a (d)NTP/NADH-linked assay (De La Cruz et al., 2000; Yadav et al., 2012). Reactions (50 μl) contained the indicated concentration of ζ toxin and the NADH enzyme mix (310 μM NADH [Roche, Germany], 100 U ml^−1^ lactic dehydrogenase [Sigma, USA], 500 U ml^−1^ pyruvate kinase [Roche, Germany], and 2.5 mM phosphoenolpyruvate [Roche, Germany]) in buffer B (50 mM Tris-HCl pH 7.5, 50 mM NaCl, 10 mM MgOAc, 1 mM DTT, 50 μg/ml BSA) with the indicated concentration of ATP, GTP or dATP, and 10 mM UNAG or uridine diphosphate-N-acetylgalactosamine (UNAGal) [Sigma, USA]. We determined the specific (d)NTPase activity (in μM) by measuring the (d)NDP production rate using a Shimadzu CPS-20A dual-beam spectrophotometer as described (Yadav et al., 2012). A standard curve with known amounts of NADH was obtained and used to convert the rate of ADP/GDP/dADP production from absorbance/time to concentration/rate (De La Cruz et al., 2000; Yadav et al., 2012).

## Results

### Toxin ζ preferentially hydrolyzes ATP

Toxin ζ hydrolyses ATP, even in the presence of a 10- to 15-fold excess of cold GTP (Tabone et al., 2014a), suggesting that toxin ζ prefers ATP to GTP (Tabone et al., 2014a). To examine these reactions, we purified toxin ζ in the absence of its cognate antitoxin ε_2_.

In the absence of UNAG, toxin ζ does not undergo autophosphorylation or hydrolyze NTP; with UNAG (2 mM) and 500 nM toxin ζ, only traces of the γ-phosphate of ATP (0.5 mM) were transferred to UNAG (Tabone et al., 2014a). We tested directly for nucleotide used preferentially by toxin ζ. Limiting ζ concentrations (60 nM) were used to analyze ζ-mediated ATP, GTP or dATP hydrolysis in physiological concentrations of UNAG and of nucleotides. The *B. subtilis* intracellular UNAG, ATP, GTP, and dATP pools approached ~10, ~10, ~5 and ~0.02 mM, respectively (Lopez et al., 1979; Lioy et al., 2012; Bittner et al., 2014).

**Figure 2:**
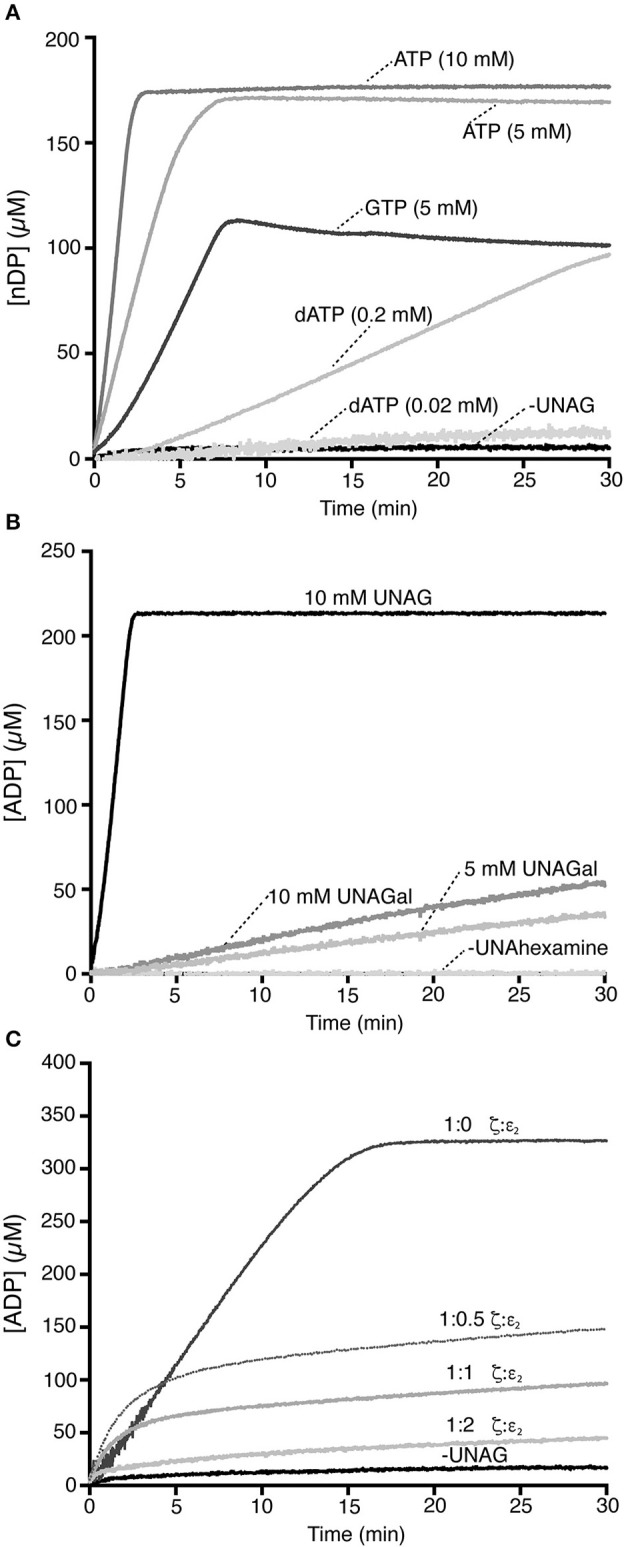
Toxin ζ preferentially hydrolyzes ATP. **(A)** A fixed toxin ζ concentration (60 nM) was incubated (30 min, 37°C) in buffer A containing 10 mM UNAG, and ATP (10 or 5 mM) or GTP (5 mM), or dATP (0.02 or 0.2 mM). The control reaction lacked UNAG. **(B)** Toxin ζ is a UNAG-dependent ATPase. Toxin ζ (60 nM) was incubated as above in buffer A with 10 mM ATP and 10 mM UNAG, or UNAGal (5 or 10 mM). **(C)** Stoichiometric ε_2_ antitoxin inhibits the UNAG-dependent ζ ATPase. A fixed ζ toxin concentration (30 nM) and increasing antitoxin ε_2_ concentrations (15-60 nM) were incubated (30 min, 37°C) in buffer A containing limiting concentrations of ATP (2 mM) and UNAG (4 mM). The amount of ATP hydrolyzed was calculated (see Section Materials and Methods). The control reaction lacks UNAG. All reactions were repeated three or more times with similar results.

Toxin ζ did not hydrolyze purine nucleotide when UNAG was omitted (Figure 2A). At physiological UNAG and ATP concentrations (10 mM each), toxin ζ (60 nM) hydrolyzed ATP in a reaction that rapidly reached saturation, which suggested that ζ is a UNAG-dependent NTPase. The final rate of ζ ATP hydrolysis approached the maximum rate (K_cat_) of 1520 ± 120 min^−1^ (Figure 2A).

The UNAG-dependent ζ ATPase activity was then compared with a *bona fide* ATPase enzyme. When the single-stranded DNA-dependent RecA ATPase was measured in parallel, *B. subtilis* RecA hydrolyzed ATP at near the previously observed K_cat_ of 9 ± 0.3 min^−1^ (Yadav et al., 2014; Carrasco et al., 2015), which suggested that ζ is a very robust ATPase. UNAG-dependent ζ-mediated ATP hydrolysis was nonetheless sensitive to variations in ATP concentration, because when ATP was reduced to half (5 mM), the K_cat_ was reduced ~3-fold (510 ± 44 min^−1^).

When ATP was replaced by physiological GTP concentrations (5 mM), ζ was able to hydrolyze GTP in a UNAG-dependent manner and the reaction reached saturation in ~7 min. The final steady state rate of GTP hydrolysis was reduced by ~5-fold (K_cat_ 280 ± 47 min^−1^) compared with physiological ATP concentrations (Figure 2A). Increasing the GTP concentration to 10 mM did not improve the reaction.

We analyzed the potential role of dATP as a substrate (Figure 2A). In the presence of physiological UNAG and dATP concentrations (10 and 0.02 mM, respectively), we observed no ζ-mediated UNAG-dependent dATP hydrolysis (Figure 2A). To test whether ζ catalyzes dATP hydrolysis, we increased its concentration artificially. At a 10-fold excess of dATP (0.2 mM), ~3 min lag time was needed to reach the steady state rate of ζ-mediated dATP hydrolysis; however, saturation was not reached in 30 min reaction (Figure 2A). With a 10-fold excess of dATP, its hydrolysis was reduced by ~20-fold compared with ATP. ATP is probably the preferred ζ nucleotide cofactor.

### UNAGal is a poor inducer of the toxin ζ ATPase

Toxin ζ interacts specifically with UNAG rather than UDP-glucose (Mutschler et al., 2011); in addition, *B. subtilis* GalE is able to interconvert UNAG and UDP-N-acetylgalactosamine (UNAGal), and the cell wall contains N-acetylglucosamine and N-acetylgalactosamine (Soldo et al., 2003). To determine whether UNAGal, a C-4 epimer of UNAG, can activate toxin ζ ATPase activity, we carried out ATPase assays with increasing UNAGal concentrations.

In the absence of UNAG or UNAGal (minus UNAhexamines), ATP hydrolysis by toxin ζ was at background level (Figure 2B). Quantitative analysis of these reactions showed that at physiological UNAGal concentrations, the final ζ-mediated ATP hydrolysis rate was ~85-fold lower (K_cat_ 20 min^−1^) than ζ in the presence of UNAG. In the presence of a UNAGal excess (10 mM), the K_cat_ was slightly increased (28 min^−1^), but was still ~60-fold lower than that at physiological UNAG concentrations (Figure 2B); this result indicates that ζ ATPase activity is specifically stimulated by UNAG rather than by UNAGal. PetZ similarly accumulates UNAG-3P after 60 min, and UNAGal-3P after 720 min incubation (Mutschler et al., 2011).

### Antitoxin ε_2_ inhibits UNAG-dependent ζ-mediated ATP hydrolysis

*In vitro*, the ζε_2_ζ complex is reported to hydrolyze ATP and phosphorylate UNAG to form inactive UNAG-3P (Mutschler et al., 2011). In contrast, *in vivo* experiments showed that the ε_2_ antitoxin inhibits the effect of toxin ζ, perhaps by forming the inactive ζε_2_ζ complex (Lioy et al., 2006, 2010). To test whether toxin ζ hydrolyzes ATP in the presence of the antitoxin ε_2_, both proteins were purified separately (Camacho et al., 2002; Tabone et al., 2014a) and UNAG-dependent ATPase activity measured.

The antitoxin ε_2_, alone or with UNAG, did not hydrolyze ATP (Figure 2C). In the presence of UNAG and ATP, the rate of UNAG-dependent ζ-mediated ATP hydrolysis was reduced by increasing antitoxin ε_2_ concentrations (Figure 2C). At ζ:ε_2_ ratios of 1:0.5 or 1:1, the kinetics of ζ-mediated ATP hydrolysis was initially unaltered, but ATP hydrolysis was inhibited after 5 min. At a slight ε_2_ excess (1:2 ratio), the antitoxin inhibited ζ ATPase activity (Figure 2C). Results were similar when both proteins were preincubated (5 min) at a 1:1 ζ:ε_2_ ratio (ζε_2_ζ complex; not shown), which suggests that when it interacts with ζ, the antitoxin occupies the ATP binding pocket (Meinhart et al., 2003) and inhibits toxin ATPase activity. This is consistent with the crystal structure of the biologically inactive ζε_2_ζ complex and with the interpretation that antitoxin ε_2_ is necessary and sufficient to inactivate toxin ζ. It is likely that the long reaction incubation time (24 h) and/or low ε_2_ stability could explain discrepancies with the previous report (Mutschler et al., 2011).

### Toxin ζ induces reversible growth arrest but a small subpopulation evades its action

**Figure 3:**
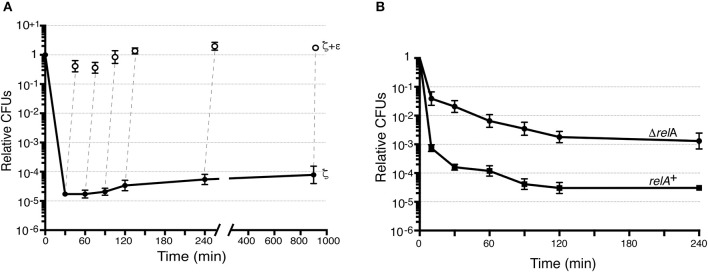
Toxin ζ induces reversible dormancy and selects for pre-existing persisters. **(A)** BG1125 cells (*lacI-P*_hsp_ ζ *spc* cassette) bearing pCB799-borne *xylR*-*P*_XylA_ε cassette were grown in MMS7 medium containing traces of xylose (Xyl; 0.05%) to ~5 × 10^7^ cells ml^−1^ (37°C). IPTG (2 mM) was added to half the culture to induce ζ expression (time 0) and the culture was further incubated. At various times, samples were withdrawn and plated in LB agar plates (•, ζ-expressing) or to allow antitoxin expression, 0.5% Xyl was added, the culture incubated (15 min) and plated onto LB agar plates (○, antitoxin ε_2_ induction). **(B)** The effect of ζY83C expression on CFU was measured. BG689 (■) or BG1145 (•) cells were cultured in MMS7 to ~5 × 10^7^ cells ml^−1^ (37°C). Xyl (0.5%) was added to half of the culture to induce ζY83C expression (time 0). At various times, samples were withdrawn and plated onto LB agar plates. Data are shown as mean ± standard error of the mean (SEM), from >4 independent experiments.

The release of toxins from their cognate antitoxins [or induction of toxin expression (+Drug in blue in Figure 1)], should lead to a bimodal time-inactivation curve if persisters appeared (dotdashed line). This deviates from the simple decay, anticipated for a population of only susceptible cells (dashed line) or for a uniformly tolerant bacterial population (dotted line, in Figure 1; Brauner et al., 2016; Harms et al., 2016). Inactivation of the toxin by expression of the antitoxin (+Drug in red) should lead to recovery of the plating efficiency (red solid line) if the toxin is bacteriostatic (Figure 1). To test whether expression of physiological levels of free toxin ζ induce persistence (dotdashed line) or tolerance (dotted line) and to study the mechanism used for such a phenotype (bacteriostasis or bacteriolysis) we performed long term survival assays. Toxin ζ was induced for a long period, and then antitoxin ε_2_ expression was induced (Figure 3A).

*Bacillus subtilis* BG1125 bearing the ζ gene under the control of IPTG induction is prone to rearrangement in the absence of IPTG (Lioy et al., 2012). To overcome this effect, the pCB799-borne ε gene under the control of Xyl was transferred into the background (Table 1; see Section Materials and Methods). BG1125 cells bearing pCB799 were grown in MMS7 supplemented with 0.05% Xyl, to ~5 × 10^7^ cells ml^−1^ (OD_560_ = 0.2), and expression of the ζ gene was induced by IPTG addition (time zero). Cells, which formed colonies after plating on LB agar without IPTG, showed a bimodal time-inactivation curve suggesting the presence of persisters (Figure 3A), rather than showing a uniform simple decay, expected for tolerant cells (Figure 1, dotted line).

To test whether the persisters are due to noise that causes instability in a bacterial population (a reduced cell fraction transiently insensitive to toxin action) or noisy gene expression (a reduced fraction with no toxin expression), we maintained IPTG induction up to 900 min, after which cells were plated in the absence of the inducer. In the former case, only a fraction of the non-replicating dormant cells would exit the arrest state and resume growth after plating without IPTG, whereas in the latter case, cell proliferation of persisters is predicted to increase 8- to 16-fold. After IPTG addition, the small persister subpopulation increased slightly (~3-fold) during the first 240 min, to later remain apparently constant (Figure 3A); this suggested negligible biological noise during the first 240 min, and persisters were transiently insensitive to toxin action.

Massive expression of toxin PezT or ζ triggers an irreversible bactericidal effect in *E. coli* grown in rich medium or *B. subtilis* grown in minimal medium, respectively (Zielenkiewicz and Ceglowski, 2005; Mutschler et al., 2011), but physiological concentrations of free toxin ζ induce a reversible bacteriostatic state (Lioy et al., 2012). To identify the source of these discrepancies, we tested whether IPTG-induced growth arrest in *B. subtilis* cells is fully reversible after antitoxin ε_2_ expression triggered by 0.5% Xyl (15 min), followed by plating in LB agar with 0.5% Xyl but lacking IPTG. Antitoxin ε_2_ expression was sufficient to reverse growth arrest, and most cells recovered proliferation, even after 900 min of toxin ζ action (Figure 3A). Although, a reduced fraction (10 to 15% of total cells) were stained with propidium iodide, suggesting a membrane compromise in these cells. It is likely that toxin ζ induces a reversible inhibition of cell growth, and that antitoxin ε_2_ expression is necessary and sufficient to switch off toxin-induced responses, with cells awakening and forming colonies even after 15 h of toxin incubation (see Figure 1, solid red line), but 10 to 15% of total cells might loss cell viability.

### Dysregulated (p)ppGpp levels increase the rate of ζY83C persisters

*Bacillus subtilis* encodes one bifunctional RelA synthase-hydrolase and two mono-functional SasA (also termed YwaC/RelP/Sas1) and SasB (YjbM/RelQ/Sas2) synthases [see Nanamiya et al., 2008; Srivatsan and Wang, 2008]. In the absence of RelA, an excess of GTP over GDP as well as baseline levels of (p)ppGpp “dysregulated” by the SasA and SasB synthases, increase toxin persistence or tolerance by >150-fold (Tabone et al., 2014b). This effect correlates with dysregulated (p)ppGpp levels. Lowering the GTP levels without affecting (p)ppGpp, by treating cells with decoyinine (a GMP synthetase inhibitor), the persistent rate was indistinguishable between treated or untreated Δ*relA* cells (Lioy et al., 2012).

To test whether the CFU increase correlates with a simple decay curve predicted from a uniform tolerant bacterial population or with a biphasic time-inactivation curve due to persistence (Figure 1), we analyzed toxin expression in the *relA*^+^ (BG689) or Δ*relA* (BG1145) cells bearing the *xylR*-*P*_xylA_ ζY83C cassette (Table 1). The *relA*^+^ and Δ*relA* cells were grown in MMS7 to ~5 × 10^7^ ml^−1^, expression of the ζY83C gene was induced with 0.5% Xyl, and the time-inactivation curve was analyzed. In the absence of RelA, a typical biphasic curve was observed upon expression of physiological concentrations of the toxin, with an ~160-fold (~5 × 10^−3^) increase in the rate of persisters after plating on LB agar without Xyl (Figure 3B).

### Varying the c-di-AMP pool alters the rate of toxin but not of Amp persistence

The second messenger c-di-AMP, which is at the heart of cell wall homeostasis, is produced mainly by Gram-positive bacteria of the phyla Firmicutes and Actinobacteria, and by some species of the δ-Proteobacteria class (Corrigan and Grundling, 2013). In Firmicutes, high or low c-di-AMP levels indirectly increase (p)ppGpp (Rao et al., 2010; Corrigan et al., 2015), whereas in *Staphylococcus aureus*, they, respectively, increase or decrease β-lactam tolerance/resistance (Corrigan et al., 2011, 2015). It is not known whether c-di-AMP has a role in toxin ζ responses to stress.

In Firmicutes, intracellular c-di-AMP levels are precisely regulated by two sets of enzymes with opposite effects and by two purine nucleotides. The diadenylate cyclases (DAC) synthesize c-di-AMP from two ATP molecules, the phosphodiesterases (PDE) degrade c-di-AMP into pApA; (p)ppGpp and pApA inhibit PDE enzyme activity (Rao et al., 2010; Corrigan and Grundling, 2013; Huynh and Woodward, 2016), which predicts that c-di-AMP levels increase during starvation. Exponentially growing *B. subtilis* cells express two DAC (DisA and CdaA) and two PDE enzymes (GdpP and PgpH; Rao et al., 2010; Corrigan et al., 2011; Corrigan and Grundling, 2013; Commichau et al., 2015; Huynh and Woodward, 2016). The absence of both DAC or of both PDE causes aberrant physiology and synthetic lethality when the medium contained high K^+^ (5 mM KCl), but one representative of each family can be deleted with no apparent effect (Corrigan et al., 2011; Corrigan and Grundling, 2013; Commichau et al., 2015; Huynh and Woodward, 2016; Gundlach et al., 2017). C-di-AMP levels vary marginally (2- to 3-fold) in cells lacking DisA, CdaA or GdpP compared to the *wt* strain (Oppenheimer-Shaanan et al., 2011; Gándara and Alonso, 2015).

To determine how *B. subtilis* cells respond to toxin-mediated stress, we induced transient toxin ζY83C expression and studied the effect of disturbing the c-di-AMP metabolic balance by deleting one DAC (DisA) or one PDE (GdpP) enzyme on an isogenic background (Table 1). In parallel, Amp was used as a second stressor at twice the MIC, in anticipation that toxin expression and Amp would respond to different physiological cues. The MIC of Amp was similar in all strains tested. After Amp exposure, a subpopulation of clonal cells yielded a biphasic time-kill curve (twodotted dashed line), which indicated that they persisted rather than becoming Amp-tolerant (dotted line; Figure 1). Similar biphasic curves are reported for other bacterial species after Amp exposure (Lewis, 2010; Amato et al., 2013; Brauner et al., 2016; Harms et al., 2016).

**Figure 4:**
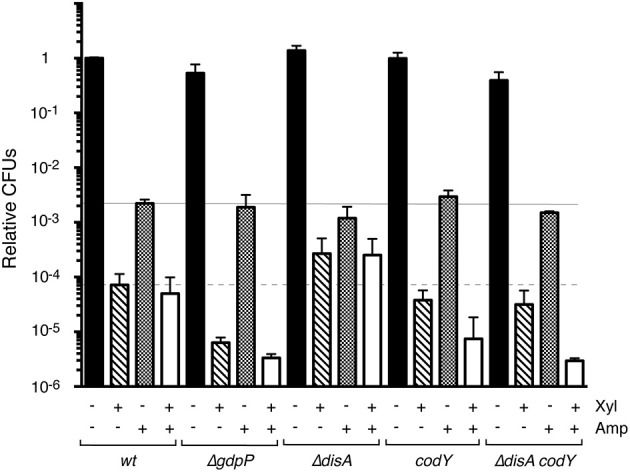
Variation in the c-di-AMP or GTP pools alters the level of ζY83C toxin persisters, but not stochastic Amp persisters during exponential growth. The set of isogenic strains was grown in MMS7 to ~5 × 10^7^ cells ml^−1^ (37°C), followed by addition of 0.5% Xyl and/or Amp (3 μg ml^−1^). Cultures were incubated (120 min), then plated onto LB agar plates. The number of CFU is shown relative to the non-induced/non-Amp-treated control. + and − denote the presence or absence of the indicated compound. Data shown as mean ± SEM, from >4 independent experiments.

*Bacillus subtilis* cells that lack GdpP show intracellular c-di-AMP levels ~2-fold higher than those of the *wt* strain (Gándara and Alonso, 2015). Absence of GdpP indirectly increases (p)ppGpp pools (Gundlach et al., 2015; Zhu et al., 2016), which suggests that a small number of specific signaling nucleotides integrate and coordinate key metabolic intersections in response to variation of the intracellular c-di-AMP pool. We constructed and analyzed a strain bearing the *xylR*-*P*_xylA_ ζY83C cassette in the context of Δ*gdpP* (Table 1). Regulated ζY83C expression in the *wt* or Δ*gdpP* contexts produced a typical biphasic survival curve, with an initial rapid decrease in CFU and a persistent subpopulation with a stable number of CFU (between 10 and 300 min; not shown); for direct comparison of the various strains, data at 120 min are shown (Figure 4). In the *wt* strain, ζY83C expression used (p)ppGpp to mediate rapid inhibition of cell proliferation, and a cell subpopulation entered a toxin- (~7.2 × 10^−5^) or Amp-persistent state (~2.1 × 10^−3^; Figure 4), as reported (Lioy et al., 2012). In the Δ*gdpP* strain, after transient toxin expression, the persistence rate decreased by ~10-fold (7 × 10^−6^), but did not significantly affect the persistence rate after Amp addition (~1.7 × 10^−3^). In the absence of GdpP, exposure to Amp and Xyl decreased the rate of persisters by ~16-fold (~3 × 10^−6^ survivals) compared to the *wt* strain (Figure 4), which suggests that a subset of toxin or Amp persisters randomly switched to the susceptible state and were targeted by Amp or the toxin.

Cells lacking DisA have ~2-fold lower levels of intracellular c-di-AMP than *wt* cells (Oppenheimer-Shaanan et al., 2011; Gándara and Alonso, 2015). We constructed the *xylR*-*P*_xylA_ ζY83C Δ*disA* strain (Table 1), and found that the persister cell rate was slightly affected by Amp addition (<2-fold, ~1.5 × 10^−3^ survivals). Transient toxin ζY83C expression increased persisters ~4-fold (~3 × 10^−4^) in the Δ*disA* compared to the *wt* strain (Figure 4). Transient toxin ζY83C expression and Amp addition did not notably affect colony formation compared to addition of Xyl alone (Figure 4).

### Absence of CodY alters the rate of toxin ζY83C but not of Amp persistence

Transient toxin ζ expression decreased the GTP pool in exponentially growing *B. subtilis* cells (Lioy et al., 2012). Intracellular GTP levels have a central role in modulating the stringent response and in reprogramming gene regulation to allow appropriate adaptation to stress. CodY, a GTP-binding protein, is a pleiotropic regulator that senses intracellular branched chain amino acids and GTP levels (Sonenshein, 2007). Low GTP levels, as found during acute stress, release CodY from DNA, leading to deregulation of genes involved in adaptation to nutrient limitation (Ratnayake-Lecamwasam et al., 2001; Belitsky and Sonenshein, 2013; Bittner et al., 2014). The role of CodY in toxin ζ stress responses is unknown. To test whether CodY modulates toxin and/or antibiotic persistence, we constructed the *xylR*-*P*_xylA_ ζY83C *codY* strain (Table 1). Lack of CodY did not markedly alter the Amp persister rate (~2.5 × 10^−3^ survivals; Figure 4). After Xyl exposure, however, we observed a slight decrease in the toxin persister rate (~2-fold) compared to *wt* cells (Figure 4). Transient Xyl and Amp addition decreased CFU (~8 × 10^−6^ survivals) and the level of persisters decreased by ~5-fold compared to the *wt* strain (Figure 4), which suggest that cells lacking CodY adapt poorly to toxin and Amp stress.

### Lack of CodY suppresses toxin persistence triggered by low c-di-AMP levels

The absence of DisA increased, and of CodY or GdpP decreased the rate of toxin persistence (Figure 4). Since lack of both CodY and GdpP strongly affected cell recovery, but the combined absence of CodY and DisA showed a less stringent phenotype, we constructed the *xylR*-*P*_xylA_ ζY83C Δ*disA codY* strain (BG1527; Table 1). The BG1527 strain yielded colonies with diffuse borders, a 3:1 normal:small size ratio, and viability reduced by ~1.4-fold compared to parental BG689 strains, but lack of CodY and DisA did not notably alter the rate of Amp persisters (~2.5 × 10^−3^ survivals). Following toxin ζY83C expression, we observed a moderate decrease (~2-fold) in the toxin persister rate (~3.5 × 10^−5^ survivals) compared to *wt* cells, similar to the *codY* strain (Figure 4). Addition of Xyl and Amp greatly decreased the persistence rate (~3 × 10^−6^ survivals; Figure 4). Different clonal subpopulations of persisting cells thus probably evolved differently in response to the toxin and Amp.

## Discussion

Toxin ζ represents a class of UNAG-dependent ATPases (Figure 2A). As another mechanism to halt cell proliferation, toxin ζ also catalyzes the transfer of part of the ATP γ-phosphate generated upon ATP hydrolysis to a fraction of UNAG, to yield unreactive UNAG-3P (Mutschler et al., 2011; Tabone et al., 2014a). Stoichiometric concentrations of purified antitoxin ε_2_ are necessary and sufficient to inactivate toxin ζ action, which suggests that no other factor contributes to ζ inactivation *in vitro*.

Using a set of isogenic *B. subtilis* strains, we tested how purine nucleotide signaling integrates and coordinates the toxin mode of action *in vivo*. Toxin ζ expression induced a biphasic time-inactivation curve with initial rapid, reversible growth arrest of the bulk of susceptible cells; a minor cell subpopulation showed non-inheritable toxin persistence rather than tolerance. Subsequent expression of the ε_2_ antitoxin reversed ζ-induced dormancy, and the cells formed colonies even after 900 min of growth arrest. After accumulation of the ζε_2_ζ complex, the heterogeneous dormancy state is nearly fully reversible; but a reduced subpopulation (up to 15%) of total cells is still stained with propidium iodide. It is likely that the ζ phosphotransferase might compromise the awakening of these cells, which may have a poor fitness or be maladapted. Persisters are formed through redundant mechanisms, and both the biological basis of persistence and the mechanisms that lead to persister formation are poorly understood in Firmicutes. Direct comparison with the well-characterized *E. coli* system could introduce some noise. For example, in both *E. coli* and *B. subtilis* cells, physiological (p)ppGpp levels are necessary for toxin-induced persistence (Korch et al., 2003; Nguyen et al., 2011; Lioy et al., 2012; Amato et al., 2013; Maisonneuve et al., 2013). In the absence of hydrolase-synthase SpoT, *E. coli* cells are not viable (Xiao et al., 1991), but in the *spoT*1 context (attenuated hydrolase activity), high levels of dysregulated (p)ppGpp give rise to hypertolerance (Amato et al., 2013; Maisonneuve et al., 2013). In *B. subtilis* cells, lack of the hydrolase-synthase RelA leads to undetectable levels of dysregulated (p)ppGpp, which in turn do not inhibit GTP synthesis and contribute indirectly to hyperpersistence (~160-fold increase). In the absence of (p)ppGpp, there is no persistence signal in *B. subtilis* or *S. aureus* cells, but reduction of GTP and ATP (Tabone et al., 2014b) or ATP levels (Conlon et al., 2016), respectively, leads to cell susceptibility to distinct antibiotics. Indeed, the artificial reduction of the GTP level sensitizes the cells to different antibiotics in the absence of (p)ppGpp (Tabone et al., 2014b). In *E. coli* cells that lack the 10 host-encoded mRNA interferases, levels of persisters to certain antibiotics decrease (Maisonneuve et al., 2011), whereas in *B. subtilis* cells, absence of the single mRNAase NdoA (MazF) increases antibiotic lethality rather than inducing persister cell formation (Wu et al., 2011).

A very small fraction of *E. coli* cells (~0.01%) is reported to have a high (p)ppGpp concentration, which triggers entry into the persistent state (Maisonneuve et al., 2013). Our study addressed the mechanism of persister formation in *B. subtilis* cells in conditions in which toxin/antitoxin expression were controlled by external inducers; antitoxin degradation thus had no role, which rendered unnecessary the analysis of (p)ppGpp in toxin release. If the stochastic switch to produce (p)ppGpp is the sole factor that triggers persister formation, the proportion of toxin and Amp persisters should be similar, and transient toxin expression and Amp addition would not further decrease cell viability. This was not observed. To explain our results, we must assume that a certain cell fraction switches stochastically to the persistent state prior to environmental challenges (Amp persisters), but sensing the metabolic state is of key importance for responsive induction of persistence. Alterations in the GTP (*codY*) or c-di-AMP pools (*gdpP* or *disA*) indicated a constant “awakening” rate after Amp addition, but a variable proportion of toxin persisters (Figure 4). Toxin ζ temporarily and reversibly increases the (p)ppGpp pool, reduces the ATP and GTP pools, and modulates c-di-AMP and UNAG levels to allow cells to readjust their metabolism from logarithmic growth to “growth arrest,” enabling cells to cope with environmental stress. The pattern of toxin persistence was varied by altering the c-di-AMP pool, which acts as two opposite mechanisms that are negatively and positively controlled by toxin expression. The subpopulation of bet-hedging persister cells that arises before changes in the environment and those triggered by toxin-induced metabolic changes both lead to toxin persisters.

Responsive strategies based on environmental sensing alter phenotypic switching between growth-arrested and persister cells. By varying the intracellular pool of signaling nucleotide, the stochastic subpopulation of toxin ζ persisters varied up to 40-fold (Δ*disA* vs. Δ*gdpP*). When both stress sources (Amp and free toxin) were present, however, a fraction of Amp (or toxin) persisters might awaken and become sensitive to the second stressor, decreasing the rate of persisters by up to 200-fold (Δ*disA* vs. Δ*gdpP* background).

Based on these results and our previous work (Lioy et al., 2006, 2012; Tabone et al., 2014a,b), we propose that the *modus operandi* of toxin ζ-induced growth arrest is to reduce the ATP (by direct hydrolysis) and GTP (by conversion to [p]ppGpp) pools. As a consequence of this, (p)ppGpp levels are increased, and a fraction of UNAG becomes phosphorylated. High (p)ppGpp directly inhibits regeneration and *de novo* GTP synthesis; it positively and negatively regulates the c-di-AMP pool, and decreases the proton-motive force (lowering the ATP pool) as well as UNAG (Kriel et al., 2012). These imbalances induce diverse transient, reversible states to ensure population survival in adverse conditions. Except (p)ppGpp dysregulation on the Δ*relA* background, there is no direct information that a discrete metabolite increases persister formation. ATP depletion is thought to be a general mechanism of persister formation in bacteria (Conlon et al., 2016; Shan et al., 2017), although we found that a reduction in ATP levels leads to ζ-induced growth arrest rather than to persister formation. We propose that the interrelationship between ATP, GTP, (p)ppGpp, c-di-AMP, and UNAG contribute, via a poorly characterized mechanism, to ζ-induced cell growth arrest and persister formation.

## Author contributions

MM, VL, MT, and JA conceived and designed the experiments for this study. MM, VL, and MT performed the experiments. JA wrote the manuscript. All authors discussed the data and made comments on the manuscript.

### Conflict of interest statement

The authors declare that the research was conducted in the absence of any commercial or financial relationships that could be construed as a potential conflict of interest. The reviewer RDO and handling Editor declared their shared affiliation, and the handling Editor states that the process nevertheless met the standards of a fair and objective review.
